# Advancing mortality rate prediction in European population clusters: integrating deep learning and multiscale analysis

**DOI:** 10.1038/s41598-024-56390-x

**Published:** 2024-03-15

**Authors:** Yuewen Shen, Xinhao Yang, Hao Liu, Ze Li

**Affiliations:** 1 School of Mechanical and Electrical Engineering, Soochow University, Suzhou, 215000 China; 2 School of Electronic and Information Engineering, Suzhou University of Science and Technology, Suzhou, 215000 China

**Keywords:** Computer science, Scientific data

## Abstract

Accurately predicting population mortality rates is crucial for effective retirement insurance and economic policy formulation. Recent advancements in deep learning time series forecasting (DLTSF) have led to improved mortality rate predictions compared to traditional models like Lee-Carter (LC). This study focuses on mortality rate prediction in large clusters across Europe. By utilizing PCA dimensionality reduction and statistical clustering techniques, we integrate age features from high-dimensional mortality data of multiple countries, analyzing their similarities and differences. To capture the heterogeneous characteristics, an adaptive adjustment matrix is generated, incorporating sequential variation and spatial geographical information. Additionally, a combination of graph neural networks and a transformer network with an adaptive adjustment matrix is employed to capture the spatiotemporal features between different clusters. Extensive numerical experiments using data from the Human Mortality Database validate the superiority of the proposed GT-A model over traditional LC models and other classic neural networks in terms of prediction accuracy. Consequently, the GT-A model serves as a powerful forecasting tool for global population studies and the international life insurance field.

## Introduction

With the continuous improvement of modern medical care and human well-being, the overall human mortality rate has been showing a decreasing trend, which is closely related to social insurance institutions, life insurance pricing and national pension payment, including current and future mortality. Time series analysis is an effective tool for capturing the evolution of mortality rates over time and will provide valuable insights into the underlying trends^1^.

Traditional time series forecasting methods utilize historical data to build mathematical models that capture features and patterns, enabling predictions of future observations. These methods include moving average, exponential smoothing, ARMA, ARIMA, SARIMA, exponential smoothing state space models, and others. Time series forecasting finds applications in economics, finance^2^, sales^3^, weather, and traffic flow prediction^4^, aiding decision-making by providing accurate forecasts for resource allocation, strategy formulation, and informed decision-making. Time series forecasting techniques play a crucial role in understanding and predicting mortality rates, these methods analyze historical data to identify patterns, trends, seasonality, cycles, and even anomalies. By decomposing these components, time series forecasting models can capture the underlying structure of how mortality rates change over time and make accurate predictions for future outcomes^5^. The widely used Lee-Carter model^6^ assumes a common stochastic trend for the evolution of age-specific mortality rates over time. Its simplicity and effectiveness have made it popular in demographic statistics and population forecasting. The model has also inspired various improvements and extensions, including the airns-Blake-Dowd (CBD) model^7^, the Booth-Maindonald-Smith (BMS) model^8^ and the Hyndman-Ullah model^9^ , and many others.

Although initially, these models are used to describe only one population, in various situations, it is useful and even necessary to model the mortality of multiple populations simultaneously. Lee^10^ argued that national mortality trends should be analyzed in a broader international context. Factors such as geographic location, transportation, and trade between countries all have an impact on individuals, making it reasonable to study mortality in the larger cluster of countries. Li and Lee^11^ suggested the use of multi-population models for mortality prediction studies, which eliminated the effect of heterogeneity by analyzing data in homogeneous clusters. Therefore, there is a growing need to develop models that capture multiple group dynamics simultaneously and incorporate a wider range of relevant factors, see for example Schnürch and Kleinow^12^, Hatzopoulosa and Haberman^13^, Cairns^14^ and Chen et al.^15^.However, commonly used human mortality data sets often contain hundreds of age-related characteristics, presenting challenges in integrating the information shared among multiple regions. Moreover, these characteristics frequently exhibit random temporal variations. Hence, a key focus of our work is to address the question of how to effectively model such complex data.

Due to the typical time series characteristics displayed in mortality data, recent techniques in time series forecasting have roots in the use of Artificial Neural Networks (ANN)^16^, which contain non-linear functions, enabling them to outperform classical algorithms^17^. In the prediction of time-series data of human mortality, neural networks have also shown excellent predictive performance. For example, Ronald et al.^18^ successfully applied the long-short-term memory (LSTM) and gated recurrent unit (GRU) in the recurrent neural network (RNN) to the Swiss population mortality time series data modeling. Wang et al.^19^ proposed a novel neighbor mortality rate prediction model that combined CNN to capture complex nonlinear structures, including neighborhood effects, surpassing classical models. Perla et al.^20^ incorporated RNN and convolutional neural network (CNN) into a network model for large-scale mortality rate prediction, which showed better predictive performance compared to the Lee-Carter model. Scognamiglio^21^ embed an individual LC model into a neural network, leveraging mortality data from all populations to jointly estimate the classical LC parameters, which have shown that neural networks enhance forecasting performance, especially for smaller populations, yielding smooth and robust parameter estimates. Perla and Scognamiglio^22^ employed Multilayer Perceptron (MLP) for large-scale mortality forecasting based on the assumption of locally-coherence of the mortality forecasts and successfully simulated mortality rates of multiple populations. Similarly, Salih et al.^23^ employed the backpropagation training algorithm to predict the number of deaths in northern Iraq, utilizing a multilayer perceptron neural network to gain insights into community characteristics and future planning. Compared to traditional mortality models, these classical neural network models demonstrate improved accuracy and robustness, highlighting their potential in mortality prediction. Therefore, in line with the studies conducted by Perla^20^ and Wang^24^, we incorporated LSTM, RNN, and 1D-CNN as comparative models for mortality rate prediction in our research.

In recent years, Transformer^25^ has achieved remarkable progress in time series prediction^26^. Its core model is good at processing end-to-end sequences and capturing long-term dependencies and interactions, leading to impressive results in time series modeling. Therefore, a large number of studies using transformers for various sequence predictions have emerged, such as power load forecasting^27^, traffic flow forecasting^28^, stock forecasting^29^, etc. In the human mortality data, the geographic information between countries is also an important feature that should not be ignored^30^. Therefore, Graph convolutional neural networks (GCN) may also have unexpected performance^31^, in time series data with spatial information. GCN is a feature extractor widely used to extract spatial information and perform prediction and classification tasks^32,33^. In the task of predicting the number of confirmed cases and deaths in COVID-19^34,35^, by selecting different adjacency matrices, GCN will extract feature information from irregular data structures within large clusters and sequences. The feature information include Euclidean and non-Euclidean distances, which are used to analyze and describe the relative positions and relationships between nodes in a graph, as well as their similarities and differences across different dimensions^36^. Therefore, for multi-cluster mortality prediction research, we propose a combined GCN and Transformer with Adaptive Adjustment Matrix (GT-A).

The main work and innovations of this research are divided into the following points: Firstly, in dealing with high-dimensional data from a single country, we utilized PCA dimensionality reduction technique to derive a unified representation that encompasses information from multiple features. To effectively integrate the low-dimensional representations of individual countries, we introduced a homogeneous clustering algorithm that explored spatial distance and non-Euclidean distance, resulting in a dynamic adjacency matrix. Furthermore, for the purpose of modeling mortality rates across multiple countries and age groups, we put forth an innovative approach that harnessed the power of graph convolutional networks to capture spatial information, along with an enhanced Transformer architecture to capture temporal sequencing.

Finally, our experiments compare our model with traditional recurrent neural networks (LSTM, RNN, 1D-CNN) and GCN-Transformer for spatio-temporal data modeling, and show that our model outperforms these traditional models.

The sections of this paper are organized as follows: In section results, we have presented a substantial volume of experimental findings, accompanied by a comprehensive comparison and analysis of these results across various dimensions. In section Numerical Application, we provide a detailed description of the experiments, including data processing, parameter setting and the construction of the adaptive matrix. In section Discussion, we summarize our findings and offer insights for future research. The Method section describes the experimental process, including time series clustering and the construction of our model framework.

## Methods

Our model GT-A (GCN and Transformer Network with Adaptive Adjustment Matrix) is introduced in this chapter, as well as the framework structure of mortality prediction model based on GCN and Transformer and the prediction principle of the model. Here is a Table 1 summarizing the meanings of certain symbols used in the paper:

**Table 1:** Definitions of the variables.

Symbol	Description
*m*	Number of countries
*n*	Optimal number of clusters
*T*	Input sequence length
$T_L$	Total sequence length
*d*	The age dimension
$A_{ada}$	Adaptive adjustment matrix
$A_{DTW}$	The similarity between the first principal components of mortality in different countries
$A_{lng-lat}$	Actual distance matrix
$X_i$	Input sample
$C_i$	the *i*-th cluster
$head_i$	The i-th attention head
*Q*	Query matrix
*K*	Key matrix
*V*	Value matrix
*softmax*	Soft version of max activation function
$d_k$	The dimension of K

### GT-A framework

**Figure 1:**
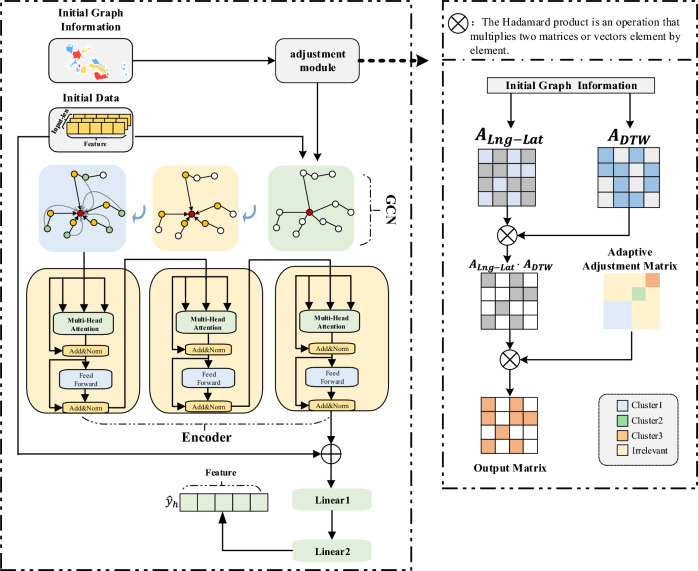
The framework of the GT-A

The framework of my GT-A model is shown in Fig. 1, which is mainly composed of two components, GCN layer and encoder layer of Transformer network. The GCN layer aims to capture the spatial information based on the relationships between different countries and the correlation information between mortality series. Then the output of the GCN layer is fed to the Encoder layer of the transformer to capture individual countries’ mortality trends and correlations over time. We utilize multiple linear layers instead of Decoder layer to further cluster spatio-temporal information and features. This design may project the hidden dimension to the desired output dimension, achieving end-to-end sequence prediction. Among them, the adjustment module adaptively adjusts the data distribution distance within the cluster through internal features, which will be described in the next section.

### GCN layers

Assume that our input mortality data is $X=(X_i,i=1,2,\ldots ,m)$, where $X_i \in \mathbb {R}^{T\times d}$, $X_i$ denotes the mortality rates in different countries, *m* is the number of countries included in the multi-cluster studied , *T* is length of time series and *d* is the dimension of ages. The input of GCN network is not only mortality data, but also an adjacency matrix $A \in \mathbb {R}^{m \times m}$ used to describe the distribution characteristics of data. The adjacency matrix A is designed to be composed of two parts multiplied by $A_{DTW} \in \mathbb {R}^{m \times m}$ and $A_{Lng-Lat}\in \mathbb {R}^{m \times m}$, where $A_{DTW}$ is the similarity between the first principal components of mortality in different countries, and calculates the Dynamic Time Warping (DTW)^37^ distances between m principal component sequences respectively. Matrix $A_{Lng-Lat}$ is the actual distance between countries, based on the latitude and longitude of each capital. In the task of realizing the simultaneous prediction of mortality in multiple countries, adding the prior conditions of the trend change of different clusters will improve the accuracy of the model^38^. Therefore, we categorize countries into the best-performing classes, as outlined in detail in section Numerical Application, and combine all countries into adjacency matrix, so that the internal relations of the same cluster are closer. We set up an adaptive adjustment matrix $A_{ada}$ to automatically adjust the correlation within clusters and between different clusters.

We define a correlation vector $[\alpha _1,\alpha _2,\ldots ,\alpha _n,\beta ]$ to help measure the correlation between national mortality data, where *n* is the optimal number of clusters, $\alpha \in [0, 1]$ under each cluster is used to reduce the distance between elements in n clusters, and $\beta \in [1,\infty ]$ is used to appropriately enlarge the distance between elements in different clusters. In Fig. 1, $A_{ada}$ has three cluster regions and one uncorrelated region. After performing the point-wise multiplication between the reference adjacency matrix and the adaptive adjustment matrix, it is placed into the model along with the original data. The propagation formula between the network layer and the layer of graph convolution neural network is as follows:

1 $$\begin{aligned} p^{l+1}=f(H^l,A)=\sigma ({\widehat{D}}^{-\frac{1}{2}} \tilde{A} {\widehat{D}}^{-\frac{1}{2}} P^l W^l +b^l),\tilde{A} =A+I , \end{aligned}$$

where $A = (A_{DTW}\cdot A_{Lng-Lat} \cdot A_{ada})\in R^{m\times m}$ (“$\cdot$” is the Hadamard product) is the adjacency matrix finally input by the model, $I\in R^{m\times m}$ is the unit matrix, and $\widehat{D}\in R^{m\times m}$ is the pairwise angle matrix of $\tilde{A}$, $P^l$ is the feature matrix of the *l*th layer, $W^l$ and $b^l$ are the weight matrix and the parametric matrix respectively.we present the detailed description of the algorithm used in our study. As shown in algorithm 1:

**Algorithm 1:**
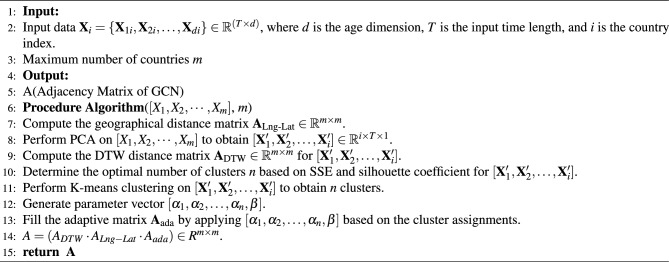
** Adaptive Adjustment Matrix.**

### Transformer layer

The Transformer model has shown remarkable ability in modeling long-term dependencies and interactions in time series data^39^. In this article, we have focused on utilizing only the encoder component of the Transformer model^40^. We adopt a sliding window with size *t* and step size *s* to predict mortality. The encoder part of the model primarily consists of a multi-headed attention mechanism, which will be expressed as follows:

2 $$\begin{aligned} Attention(Q,K,V) = softmax\left( \frac{QK^{T}}{\sqrt{d_{k}}}\right) V, \end{aligned}$$

where $Q\in \mathbb {R} ^{T \times d_{model}}$ is query item matrix, $K\in \mathbb {R} ^{T \times d_{model}}$ is key item matrix ,$V\in \mathbb {R} ^{T \times d_{model}}$ is value item that needs to be weighted averaged. The *Q*, *K*, *V* matrix are obtained by multiplying each input vector of the encoder by three weight matrices $W_{q}\in \mathbb {R}^{d_{model} \times d_{k}}$, $W_{k}\in \mathbb {R}^{d_{model} \times d_{k}}$, $W_{v}\in \mathbb {R}^{d_{model} \times d_{v}}$, where $d_k=d_v={d_{model}}/h$. $W_q$, $W_k$, $W_v$ are the parameters that the network needs to learn and train. The *Q* vector and the *K* vector are multiplied to obtain the attention score and determine the attention distribution. The standardized attention scores are compared with each multiplied *V* vector, which are obtained by passing the attention scores through the softmax layer. The higher the score, the greater the multiplied value, and the more attention it receives. The following equation represents the formula for the multi-headed attention mechanism:

3 $$\begin{aligned} \begin{aligned} \text {MultiHead}(Q,K,V)&=\text {Concat}(head_{1},\cdots ,head_{h})W_o,\\ head_{i}&=\text {Attention}(QW_{i}^{Q},KW_i^{K},VW_i^{V}). \end{aligned} \end{aligned}$$

## Numerical application

### data

**Figure 2:**
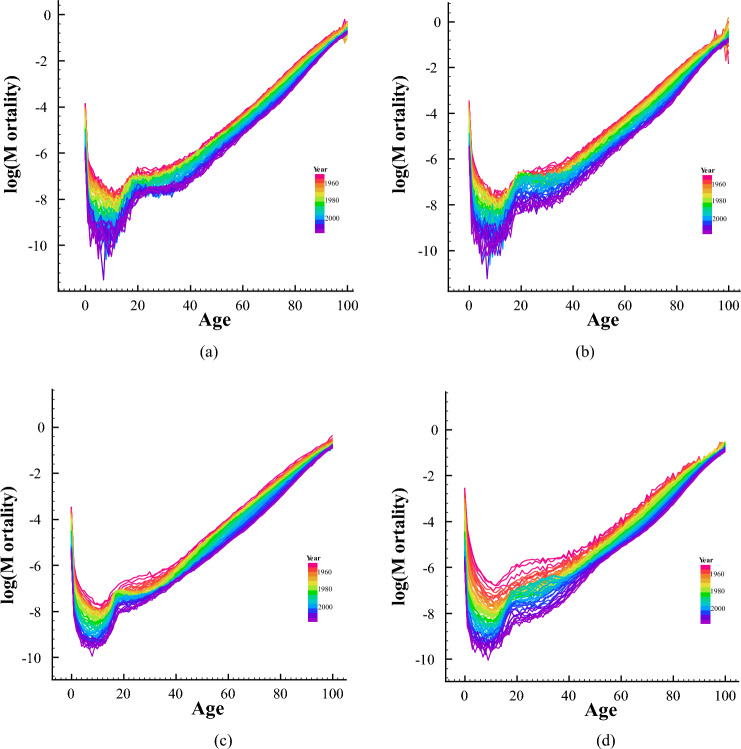
Log-mortality rates over time for each age in four countries.

Referring to the benchmark papers we selected^20^, we select the mortality data of 16 European countries with the satisfaction time of $1950\le T_L\le 2016$ and the age of $0\le d\le 100$ from the human mortality database^41^. This is also consistent with the suggestion given by the Human Mortality Database that the data after 1950 is relatively stable because the Second World War ended in 1945. Mortality data during the world wars are not informative. In the mortality database, there are 41 countries. Among them, 16 European countries meet the span of 1950-2016. During data preprocessing, we take the average mortality rate of all countries at the same time and age instead of missing values. In order to train and evaluate our model, the proposed model is trained from 1950 to 2000 and the mortality from 2001 to 2016 is predicted. Figure 2 shows the natural logarithmic mortality trend of four selected countries: Sweden in northern Europe, Switzerland in central Europe, Britain in western Europe and Spain in southern Europe. Due to the limitation of time span, eastern European countries are not included.

As shown in the Fig. 2, with the passage of time, the mortality rate of European countries in the four different geographical regions has generally shown a downward trend, while countries from different geographical regions have their own unique mortality curve characteristics. Therefore, by clustering and analyzing the mortality rate of the 16 European countries, we group them into different clusters and assign the same adaptive parameters to the countries within each cluster. This approach helps determine the distribution of adaptive parameters in the matrix $A_{ada}$.

### Adjacency matrix

The input adjacency matrix *A* of GCN is introduced in this section, which is obtained by multiplying three matrices $A_{ada}$, $A_{DTW}$ and $A_{Lng-Lat}$, in which $A_{DTW}$ and $A_{Lng-Lat}$ are obtained by calculating the DTW distance and geographical distance between countries respectively. For the distribution of adaptive parameters in the adaptive adjustment matrix $A_{ada}$, we get the best number of clusters by analyzing and clustering the data, and give the same adaptive parameters as the countries in the same cluster.

#### Dynamic time warping

To measure the similarity between sequences, the DTW algorithm is employed, which calculates the distance between two sequences as a measure of their similarity. The steps of the DTW algorithm are as follows: Firstly, calculate the distance matrix between the points of two sequences. Secondly, find a path from the upper left corner to the lower right corner of the matrix to minimize the sum of elements on the path. Finally, the distance between two time series *A* and *B* is the sum of the minimum values of all possible paths.

Defining two time sequences of length *n* as *A* and *B*, $M(i,j)=\left| A(i)-B(j) \right|$ is the distance matrix between the two sequences , where $i\ge 1$, $j\le n$. In the distance matrix, the path length from the top left corner to the bottom right corner is equal to sum of the path length of its step and the current element size, where the previous element of the element $a_{i,j}$ on the path should be $b_{i,j-1}$, $c_{i-1,j}$, $d_{i-1,j-1}$. Then in the recursive algorithm, the minimum value $L_{\min }(i,j)$ of the cumulative distance will be expressed as follows:

4 $$\begin{aligned} L_{\min }(i,j)=\min \{L_{\min }(i,j-1), L_{\min }(i-1,j),L_{\min }(i-1,j-1)\}+M(i,j). \end{aligned}$$

#### PCA analysis of age dimension in each country

Before clustering the data, we use principal component analysis (PCA) to reduce the dimension. The mortality series of each country is composed of several age groups, and each time point has multidimensional variables, so it is necessary to replace the overall variables with a few variables from the multidimensional time series, while retaining most of the information in the data. For individual country mortality data $X_i=(x_{1i},x_{2i},\ldots ,x_{di})^T \in \mathbb {R}^{T \times d}$ where *T* is length of time series and *d* is the dimension of ages, *i* is the *i*th country. So that the mean vector is $\mu _i=E(X_i)=( \mu _{1i}, \mu _{2i},\ldots ,\mu _{di})^T$, and the covariance matrix $\Sigma =Cov(X_i,X_i)=E[(X_i-\mu _i)(X_i-\mu _i)^T]$. Considering the linear transformation of the d-dimensional variable $X_i$ to the variable $y_i=(y_{1i},y_{2i},\ldots ,y_{di})^T$:

5 $$\begin{aligned} y_j={\alpha _j}^T X_i= \alpha _{1j}x_{1i}+ \alpha _{2j}x_{2i}+\cdots +\alpha _{dj}x_{di}, \end{aligned}$$

where ${\alpha _j}^T = (\alpha _{1j},\alpha _{2j},\ldots ,\alpha _{dj})$, $j=1,2,\ldots ,d$. The variable $y_{1}$ corresponds to the linear transformation of $X_i$ with the largest variance. By calculating the covariance matrix of $X_i$, the first principal component of $X_i$ is obtained. The proportion of variance explained by the first principal component for each country will be found in the following Table 2:

**Figure 3:**
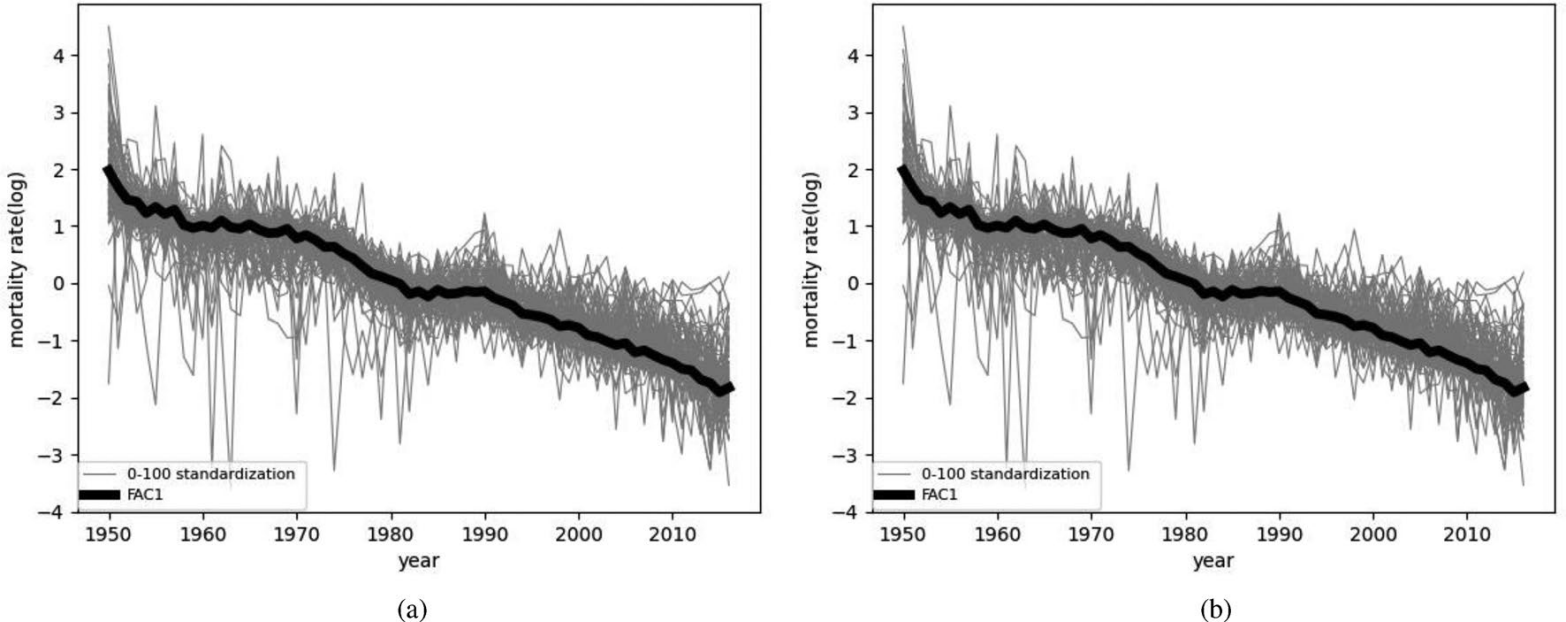
Trends in mortality and standardization of data.

**Table 2:** Proportion of four principal components in 16 countries (%).

Main Ingredient	**FAC1**	FAC2	FAC3	FAC4
Finland	**79.408**	5.872	1.337	1.120
Denmark	**94.662**	2.016	1.022	0.763
France	**86.161**	2.390	1.371	1.149
Netherlands	**90.323**	3.396	0.879	0.647
Norway	**80.498**	3.536	2.597	1.045
Sweden	**87.648**	2.679	1.095	0.977
Spain	**89.589**	4.062	2.525	1.124
Belgium	**89.285**	2.424	1.481	0.833
U.K.	**91.722**	3.879	1.429	0.699
Switzerland	**87.392**	3.458	1.227	0.921
Italy	**94.865**	2.167	0.949	0.270
Austria	**90.767**	1.407	1.063	0.908
Portugal	**87.836**	4.398	1.875	1.081
Hungary	**62.557**	24.839	2.027	1.417
Slovakia	**66.364**	13.854	3.301	1.157
Czech	**85.880**	3.898	1.460	1.169

Significant values are in bold.

In PCA, the first principal component height represents the overall trend of mortality change. Therefore, we chose the first principal component for cluster analysis, taking Denmark and Finland as examples. As shown in Fig. 3, gray represents 101 standardized mortality curves that change with time, and the thick black line represents the first principal component, which clearly describes the overall trend of mortality changes.

#### Shape-based time series clustering with adjacency matrix construction

**Figure 4:**
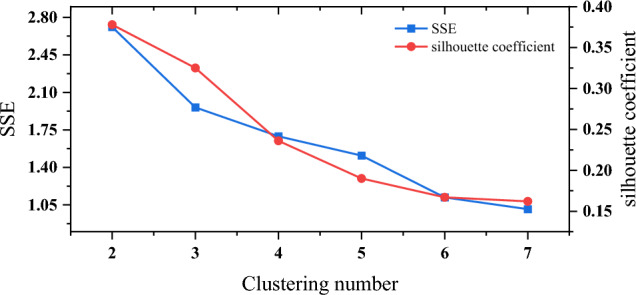
First principal component clustering profile coefficients and SSE.

After measuring the distance or similarity between samples using metrics, we utilize the first principal component of the 16 countries mentioned above for DTW clustering. We choose K-means clustering method to cluster samples, which randomly selects *k* center points in *k* clusters. Then the center of each cluster is calculated by iteration, and samples are distributed according to the distance. The expressions for this algorithm are as follows:

6 $$\begin{aligned} \begin{aligned} \text {Minimize squared error:} E&= \sum _{i=1}^k \sum _{x\in C_i} i {\Vert x-{\mu }_i \Vert _2}^2,\\ \text {Centroid:} {\mu }_i&=\frac{1}{|C_{i} |}, \end{aligned} \end{aligned}$$

where $C_i$ is the *i*-th cluster. As the evaluation index of clustering, the contour coefficient is chosen, and the calculation formula is as follows:

7 $$\begin{aligned} \begin{aligned} S(i)&=\frac{b(i)-a(i)}{\max \{a(i),b(i)\}},\\ a(i)&=\frac{1}{n-1} \sum _{j\ne i}^n {\text {distance}(i,j)}, \end{aligned} \end{aligned}$$

where *j* represents the other sample points within the same category as sample *i*. The value of *b*(*i*) needs to traverse other cluster groups to get $\{b_1(i),b_2(2),\ldots ,b_m(i)\}$. In addition, we introduce the sum of squared errors (SSE): $SSE=\sum _{i=1}^{k}\sum _{p\in C_{i}}^{} \left| p-m_{i} \right| ^{2}$, in order to help evaluate the clustering criteria. Figure 4 displays the Silhouette Coefficient and SSE values obtained from clustering the first principal component.

**Figure 5:**
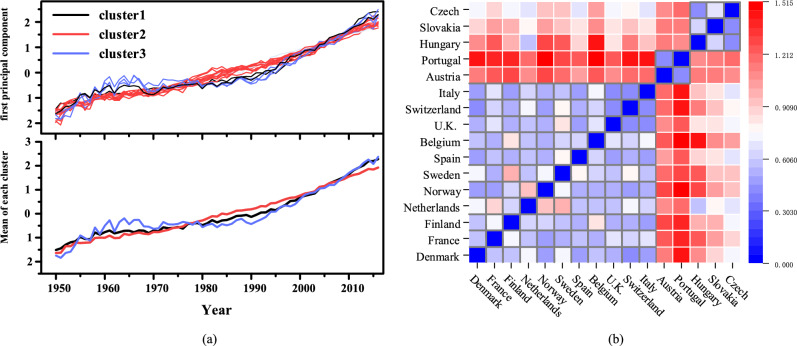
Mortality first principal component cluster analysis.

According to Fig. 4, we choose $n=3$ as the optimal number of clusters. We plot the clustering results in Fig. 5, where France, Finland, Netherlands, Norway, Sweden, Spain, Belgium, Italy and Austria are clustered into the first category; Portugal, Britain, Switzerland, Italy and Austria belong to the second category; Denmark, Slovakia and the Czech Republic are clustered into the third category.

## Results

In this chapter, we evaluate the effectiveness of our model through experiments, which are divided into two steps. The first step is to verify the performance of our model under the same large cluster forecast, and the second step is to compare our large cluster forecast results with those of other models in a single country, so as to verify that the prediction results of our model in a single country are equally effective.

### Large cluster prediction results

According to the mortality data of our 16 countries, we set $m= 16$ and $T=16$, which indicates that the forecast time is 16 years. In order to compare the effects of our models, we choose the classic multivariate time series prediction models, namely LSTM, 1D-CNN and RNN. In our model training, we fixed the epoch to 500 for each model and used the Adam optimizer with the mean squared error (MSE) loss function. The initial learning rate is set to $lr=0.001$. To evaluate the performance of the models, we calculate the root mean squared error (RMSE) for different time periods, age groups, and overall predictions^42^. In addition to the RMSE metric, we also utilized the MAE and MAPE metrics to calculate overall performance for both dimensions. The evaluation indicators used are as follows:

8 $$\begin{aligned} \begin{aligned} RMSE_{\text {all}}&=\sqrt{\frac{1}{m \times T \times d} \sum _{i=1}^{m} \sum _{t=1}^{T} \sum _{a=1}^{d}\left( y_{i, t, a}-\widehat{y_{i, t, a}}\right) ^{2}},\\ MAE_{\text{ all } }&=\frac{1}{m \times T \times d} \sum _{i=1}^{m} \sum _{t=1}^{T} \sum _{a=1}^{d}\left| y_{i, t, a}-\widehat{y_{i, t, a}}\right| ,\\ MAPE_{\text{ all } }&=\frac{100\%}{m \times T \times d} \sum _{i=1}^{m} \sum _{t=1}^{T} \sum _{a=1}^{d}\left| \frac{y_{i, t, a}-\widehat{y_{i, t, a}}}{y_{i, t, a}} \right| ,\\ RMSE_{\text {all}}(t)&=\sqrt{\frac{1}{m \times d} \sum _{i=1}^{m} \sum _{a=1}^{d}\left( y_{i, t, a}-\widehat{y_{i, t, a}}\right) ^{2}}.\\ \end{aligned} \end{aligned}$$

$RMSE_{\text {all}}$, $MAE_{\text {all}}$, $MAPE_{\text {all}}$ measure the prediction error of all countries, and the values calculated from the forecast values of each model are shown in Table 3. The results demonstrate that our model is superior to other series forecasting models in large cluster forecasting. In addition, the GT-A model with the adaptive adjustment matrix shows improvement compared to the GT model, which proves the effectiveness of our proposed adaptive adjustment matrix.

**Table 3:** Summary of $RMSE_{\text {all}}$, $MAE_{\text {all}}$, $MAPE_{\text {all}}$ over country groups.

Model	LSTM	RNN	CNN	TF	GT	GT-A
${RMSE_{\text {all}}}$	0.2202	0.2403	0.2192	0.2117	0.1860	**0.1729**
${MAE_{\text {all}}}$	0.1359	0.1423	0.1529	0.1373	0.1246	**0.1042**
${MAPE_{\text {all}}}$	2.6162	2.6739	3.2913	2.8139	1.4750	**1.1941**

Significant values are in bold.

**Figure 6:**
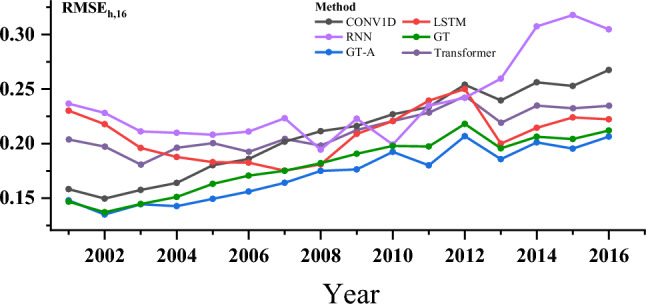
$RMSE_{\text {all}}(t)$ over forecasting steps of all conutries.

Figure 6 shows the $RMSE_{\text {all}}(t)$ values of different models from 2001 to 2016. With the passage of time, the RMSE values of all models gradually increase. Specifically, 1D-CNN, GT and GT-A show relatively stable performance, while LSTM and RNN show oscillation. As well as the latest references in the field of human mortality prediction using deep learning, as shown in the references^24^, we used the transformer model(TF) with the same parameters as a preliminary comparison. Among all the models, GT and GT-A perform best in the step size prediction task, and GT-A performs better than GT.

**Figure 7:**
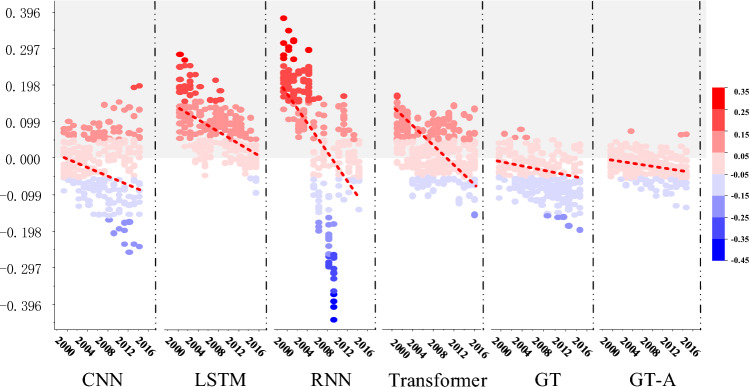
The prediction error of all countries in the six models in the time dimension.

**Figure 8:**
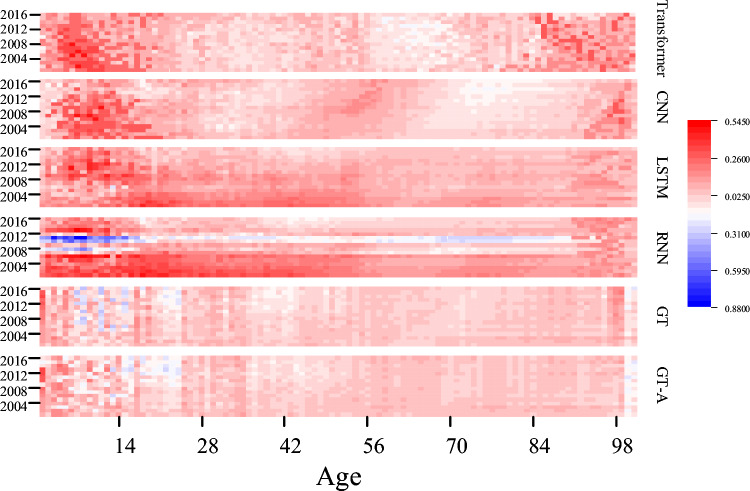
The prediction error of all countries in the six models in the age dimension.

Figures 7 and 8 show the residuals of predicted values and true values of all countries in the six models in the time dimension and the age dimension respectively, in which red indicates overestimation and blue indicates underestimation. In Fig. 7, the red dotted line represents the linear fitting curve of scattering points and the overall trend of scattering errors. The errors of LSTM ,RNN and Transformer tend to be overestimated, while the errors of 1D-CNN, GT and GT-A are relatively smooth in overall error, and the slopes of GT and GT-A are the lowest. Figure 8 presents the heat map of the average mortality error by age in 16 countries. The prediction error is obtained by subtracting the average predicted value from the average true value and then subtracting the estimated standard deviation of each age group.

All models exhibit inhomogeneities in error oscillations at low ages, caused by the excessive transition differences between unstable mortality at low ages and stable mortality at middle and low ages. Therefore, mortality prediction in the lower age groups requires higher performance of the model. Figure 8 shows that 1D-CNN, LSTM, RNN,Transformer have large overestimated parts between low to middle and low ages. In contrast, although GT and GT-A models present oscillatory irregular regions at low ages, the overall residuals are low. In the middle age region, GT and GT-A models perform evenly without abrupt overestimation and underestimation, especially from around age $30\le d\le 90$, which is the best performance. The higher age range exhibits areas of irregularity, similar to those observed in the lower age range. The GT-A model shows a small improvement compared to the GT model at low and high ages and overall has lower mean mortality error. The results demonstrate that our model outperforms traditional time series prediction models over a large cluster range, which indicate that additional spatial information, namely homogeneous information, improves prediction accuracy in the processing of large cluster data. Our model not only improves the performance of simultaneous prediction but also obtains better performance in single country forecasting.

### Experiment of comparing the prediction results

For the prediction results of a single country, we define the following indicators to measure the prediction performance of different models:

9 $$\begin{aligned} \begin{aligned} RMSE&=\sqrt{\frac{1}{ T \times d} \sum _{t=1}^{T} \sum _{a=1}^{d}\left( y_{t, a}-\widehat{y_{t, a}}\right) ^{2}},\\ MAE&=\frac{1}{ T \times d} \sum _{t=1}^{T} \sum _{a=1}^{d}\left| y_{t, a}-\widehat{y_{t, a}}\right| ,\\ MAPE&=\frac{100\%}{ T \times d} \sum _{t=1}^{T} \sum _{a=1}^{d}\left| \frac{y_{t, a}-\widehat{y_{t, a}}}{y_{t, a}} \right| . \end{aligned} \end{aligned}$$

**Figure 9:**
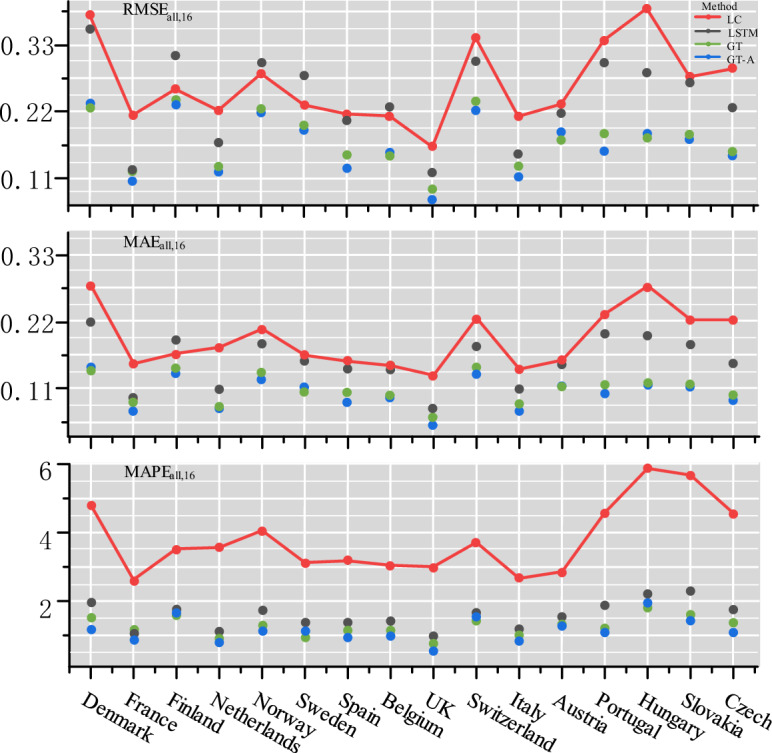
Comparative analysis of prediction-related indicators among 16 countries from 2001 to 2016.

In Fig. 9, performance metrics are calculated for all age groups and time horizons across 16 countries, and it is evident that the GT and GT-A models outperform the LC model in all countries. Specifically, in terms of RMSE, GT and GT-A outperformed the LC model by 52.4% and 59.2%, respectively. The following formula represents the percentage calculation method, where *m* denotes the total number of countries included in the calculation. The index of the i-th country under *model*1 is denoted as $RMSE_i(model1)$. In terms of MAE, GT and GT-A exceeded the LC model by 72.2% and 79.2%, respectively. Finally, in terms of MAPE, GT and GT-A exceeded the LC model by 206.4% and 223.6%, respectively. The countries with larger values across all metrics may be attributed to the small size of their population. For instance, the populations of the top three countries with the highest indicators, namely Denmark, Hungary, and Slovakia, are 5.911 million, 9.689 million, and 5.430 million, respectively.

10 $$\begin{aligned} \begin{aligned}{}&RMSE_{mean1}=\frac{1}{m} \sum _{i=1}^{m}RMSE_i(model1),\\&RMSE_{mean2}=\frac{1}{m} \sum _{i=1}^{m}RMSE_i(model2),\\&Percent=(( RMSE_{mean1}-RMSE_{mean2})/RMSE_{mean1})*100\%. \end{aligned} \end{aligned}$$

In Table 2, we have selected six representative countries from Northern, Western, Central and Southern Europe for specific analysis, with the calculation based on the age group of each country. The optimal performance is indicated by bold markings. As shown in Table 4, both the GT model and the GT-A model outperform the LC model across all six indicators. Moreover, the GT-A model with the addition of the adaptive control matrix outperformed the GT model in 83.3% of the indicators.

**Table 4:** Predictive performance of four models in different countries.

Model	RMSE^a^	MAE^a^	MAPE^a^	Mean^b^	$\mathbf {Q_1}$^c^	$\mathbf {Q_3}$^d^
UK						
LC	0.1630	0.1320	3.0010	0.1460	0.1927	0.1114
LSTM	0.1215	0.0830	0.8439	0.1024	0.0639	0.1248
GT	0.0942	0.0648	0.7236	0.0719	**0.0336**	0.1574
GT-A	**0.0750**	**0.0512**	**0.5710**	**0.0682**	0.0355	**0.0592**
France						
LC	0.2150	0.1510	2.6180	0.1650	0.0704	0.2928
LSTM	0.1255	0.0934	0.9668	0.1113	0.0675	0.1417
GT	0.1260	0.0895	1.1069	**0.0898**	**0.0673**	0.3223
GT-A	**0.1074**	**0.0735**	**0.9037**	0.0913	0.0696	**0.0742**
Italy						
LC	0.2130	0.1420	2.6580	0.1600	0.1130	0.1855
LSTM	0.1507	0.1071	1.0447	0.1285	0.0721	0.1749
GT	0.1313	0.0899	0.9584	0.0987	0.0572	0.1402
GT-A	**0.1122**	**0.0750**	**0.7596**	**0.0897**	**0.0563**	**0.0517**
Spain						
LC	0.2170	0.1550	3.1700	0.1770	0.0536	0.3206
LSTM	0.2063	0.1436	1.2722	0.1683	0.0681	0.2420
GT	0.1516	0.1057	1.0853	0.1170	0.0547	0.1270
GT-A	**0.1244**	**0.0847**	**0.8647**	**0.1010**	**0.0486**	**0.0676**
Sweden						
LC	0.2320	0.1650	3.1270	0.1897	0.1666	0.1890
LSTM	0.2803	0.1563	1.2730	0.1906	0.0649	0.2103
GT	0.1957	**0.1103**	**0.9958**	0.1391	0.0539	0.1572
GT-A	**0.1934**	0.1129	1.0982	**0.1352**	**0.0489**	**0.1494**
Switzerland						
LC	0.3430	0.2260	3.7180	0.2616	0.0858	0.4345
LSTM	0.3043	0.1802	**1.4848**	0.2181	0.0850	0.2449
GT	0.2377	0.1464	1.4929	0.1838	0.0890	**0.2409**
GT-A	**0.2236**	**0.1367**	1.5796	**0.1716**	**0.0659**	0.2531

^a^*RMSE*,*MAE*,*MAPE* are the overall RMSE,MAE,MAPE across all ages and time horizons in each countries.

^b^Mean is the sample mean of the RMSEs over age groups.

^c^$Q_1$,$Q_3$ are the first quartile, and third quartile of the RMSEs over age groups.

^d^GT,GT-A are the GCN-Transformer model and the GCN-Transformer model with the addition of adaptively adjusted adjacency matrix, respectively.

Significant values are in bold.

**Figure 10:**
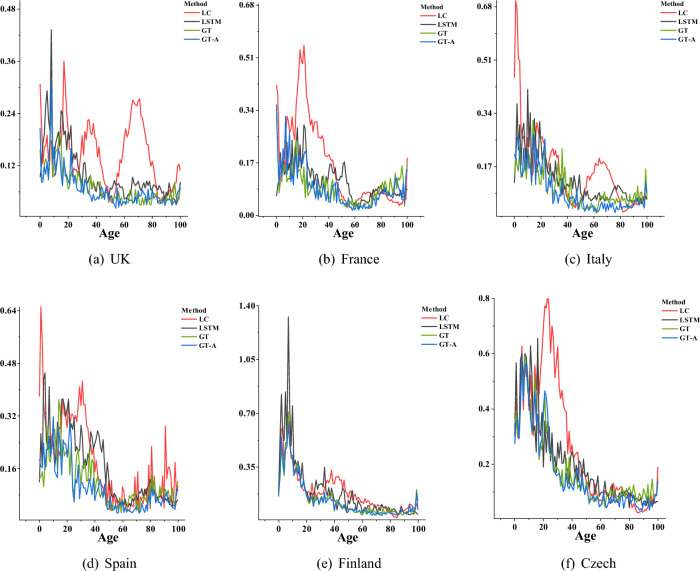
RMSE over forecasting years.

Based on Fig. 10, it is observed that the LC model performed poorly in mortality prediction in young age as well as in young adulthood for the UK and Czech, with severe oscillations in RMSE. In contrast, the RMSE curves of GT and GT-A are much smaller than that of the LC model between the ages of 10 and 50 years, showing more stability in performance. The RMSE curves of GT-A are generally lower than those of GT during middle age to old age. For instance, the GT-A model performed significantly better than GT within $60\le d\le 85$ in Italy, Finland, and Czech. Overall, the GT-A composite model exhibited the greatest advantage in the entire age group.

**Figure 11:**
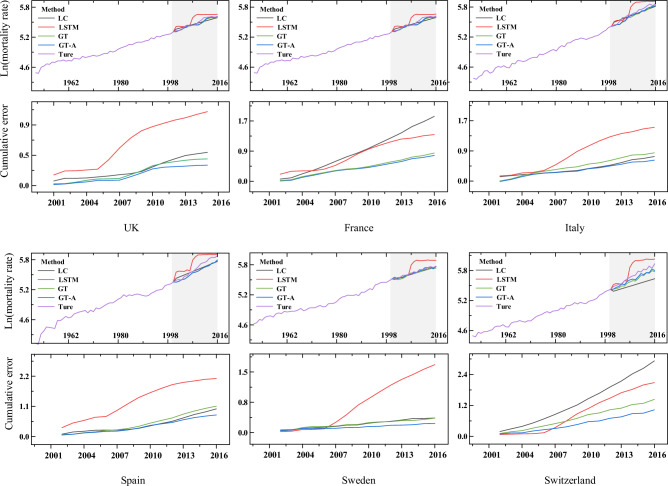
Forecast versus actual mortality rates averaged across $0\le d\le 100$ and prediction error.

To visually compare the predicted values with the true values, we follow the approach of Li and Lu^42^ and plot the average mortality rates for ages $0\le d\le 100$. The plot includes the true average mortality rates from 1950 to 2016 and the average predicted mortality rates for the three models from 2001 to 2016. As shown in Fig. 11, the LC model exhibits a flat curve in the 16-year average forecast value from 2001 to 2016, which fails to capture the fluctuation change of the average true value. In contrast, the GT and GT-A models are able to fluctuate with the fluctuation of the true value, and in most countries, the average forecast value of GT-A is closer to the average true value. Through the analysis of the cumulative error, it becomes evident that the error of the LC model experiences a rapid increase, in contrast to the other three models which demonstrate a more moderate performance. The GT and GT-A models consistently exhibited the highest level of performance across most countries.

RMSFE (Root Mean Squared Forecast Error) can be utilized to assess the performance of our predictions, which bears similarities to RMSE (Root Mean Squared Error). Once we have obtained the RMSFE, it enables us to compute prediction intervals. These intervals represent the range within which we expect our predicted values for future observations to fall. Typically, prediction intervals are expressed as intervals that encompass the likely range of true observations.

**Figure 12:**
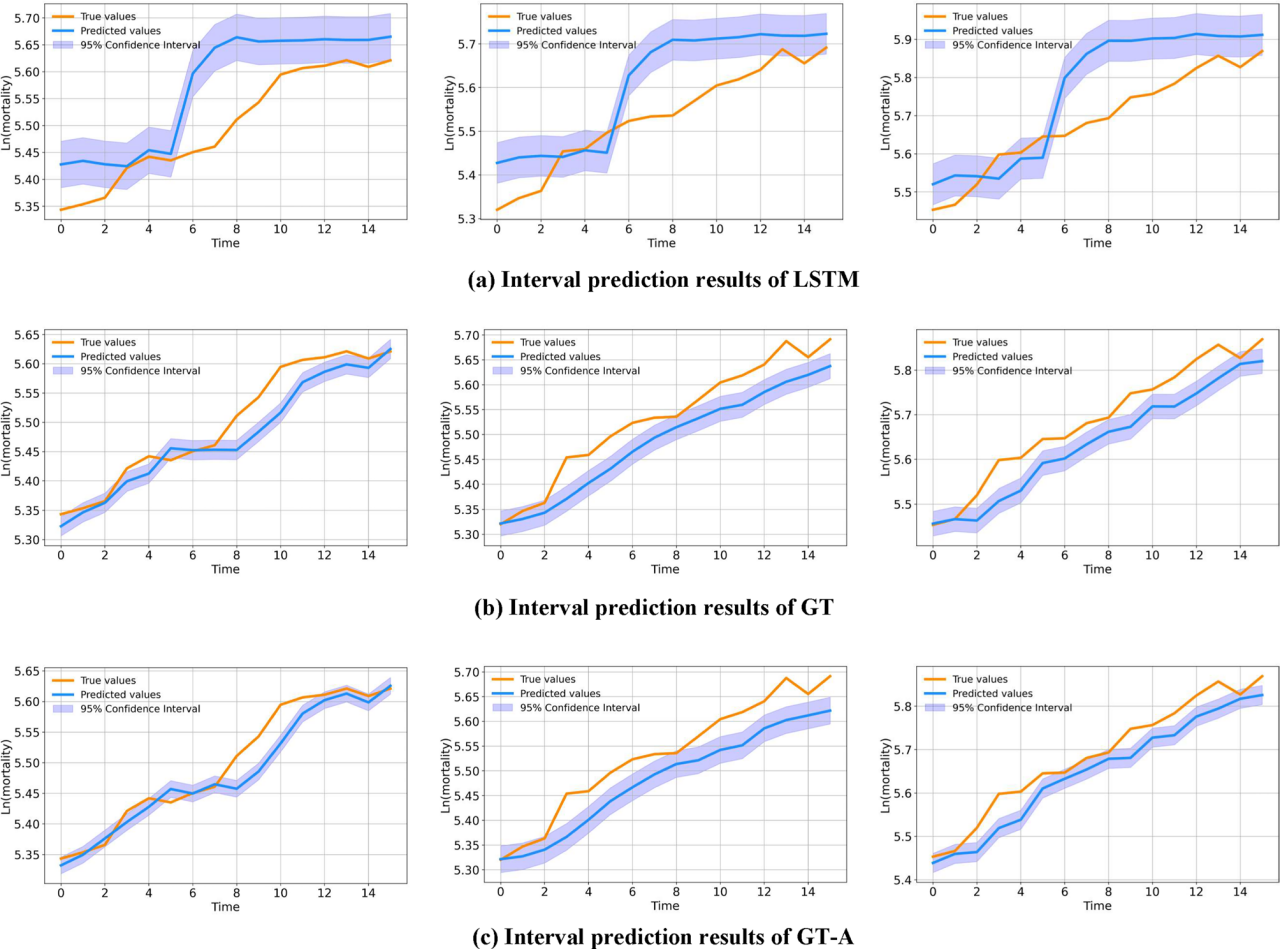
Comparison of interval prediction results for three models.

The width of the prediction interval is typically determined by the confidence level and the forecast error (RMSFE). The confidence level denotes the desired probability of the prediction interval encompassing the true observation. For instance, selecting a confidence level of 95% indicates that we aim for a 0.95 probability of the prediction interval covering the true observation as shown in Fig. 12.

In a normal distribution, around 95% of the observations lie within two standard deviations of the mean. Hence, leveraging the properties of the normal distribution, we can employ the RMSFE and confidence level to calculate the size of the prediction interval.

## Discussion

In this study, we present a novel model GT-A to simultaneously predict mortality for multiple countries in large clusters, representing a major improvement in mortality prediction models for large clusters. By incorporating spatial information and similarity information between multidimensional series, the traditional task of mortality time series prediction is efficiently incorporated into spatial data for the first time. The results demonstrate that exploiting similarity information between multidimensional series to capture spatial location information in large clusters has the ability to effectively improve the accuracy of time series forecasting. In large-scale cluster experiments, the prediction performance of the GT-A model is better than that of the traditional time series prediction model. Furthermore, the addition of the adaptive adjustment matrix of clustering information improves the regional heterogeneity in large clusters, and enhances the accuracy of the model to some extent. These findings suggest that future research should be conducted on the heterogeneity existing in each dimension in the study of large clusters to further improve prediction accuracy.We also find that simultaneous prediction for large clusters will improve prediction accuracy of a single national data set (Supplementary Information). Compared with the traditional LC model with single data set prediction, GT-A achieves better performance across the board. As Li and Lee^11^ said, it was possible to improve the prediction accuracy of mortality of a single population by capturing mortality trends common to several populations with higher statistical confidence.So far, there are various variants of the Transformer model. However, the mortality prediction model has not progressed synchronously, so this study will innovatively apply Transformer to mortality prediction and combine GCN network to capture geographic information. Furthermore, we also consider exploring the design of more suitable models for mortality rate prediction in future research to enhance its accuracy. For instance, combining attention mechanisms with the lc model could be a potential approach. This is because advancements in mortality rate prediction models have the potential to greatly benefit public health initiatives and decision-making processes. Moreover, the black box nature of neural networks presents uncertainty that prevents a full understanding of the mathematical and actuarial principles behind them, which is also the field of future research and development.

### Supplementary Information

Supplementary Information.

Supplementary Information.

## Data Availability

The datasets generated and/or analysed during the current study are available in the [Human Mortality Database] repository, [https://mortality.org/].
