# Supplementary material for: Advancing mortality rate prediction in European population clusters: integrating deep learning and multiscale analysis
Source: Sci Rep. 2024 Mar 15;14:6255. doi: 10.1038/s41598-024-56390-x (PMC10942990; doi:10.1038/s41598-024-56390-x)
Supplement: Supplementary file 2 — Supplementary Information. [file 41598_2024_56390_MOESM2_ESM.docx]

The Human Mortality Database (HMD:https://mortality.org/) is the world´s leading scientific data resource on mortality in developed countries. The HMD provides detailed high-quality harmonized mortality and population estimates to researchers, students, journalists, policy analysts, and others interested in the human longevity. The HMD follows open data principles.

The HMD strives to include all populations for which death registration and census data are virtually complete. As a result, countries and areas in the HMD are relatively wealthy and for the most part highly industrialized. At present the database contains detailed population and mortality data for 37 developed countries and 46 populations (including sub-national groups), with series starting as early as 1751 in Sweden and covering more than a 100 years for 16 populations. The selection criteria for inclusion of a country in the HMD rely mainly on the quality of its data. In order to be included in the HMD, a country’s death registration system must be nearly complete (close to 99%). For such countries, original statistics are then collected extending as far back in time as there are age-classified census data and annual death counts in a sufficiently detailed format (at a minimum, there should be 5-year age groups with a separate category for infants and an open interval at age 80 or higher). Note that for historical data (especially for years preceding 1870–80), the quality is often lower, especially at ages 80 and above.

In this paper, we select the mortality data of 16 European countries with the satisfaction time of 1950 ≤ T ≤ 2016 and the age of 0 ≤ d ≤ 100 from the human mortality database, Including Denmark, France, Finland, Netherlands, Norway, Sweden, Spain, Belgium, UK, Switzerland, Italy, Austria, Portugal, Hungary, Slovakia, Czech. During data preprocessing, we take the average mortality rate of all countries at the same time and age instead of missing values.
